# Quality of Life Theory II. Quality of Life as the Realization of Life Potential: A Biological Theory of Human Being

**DOI:** 10.1100/tsw.2003.83

**Published:** 2003-10-13

**Authors:** Soren Ventegodt, Joav Merrick, Niels Jorgen Andersen

**Affiliations:** The Quality of Life Research Center, Copenhagen K, Denmark

**Keywords:** Quality of Life, QOL, human development, holistic medicine, public health, Denmark, etiology

## Abstract

This review presents one of the eight theories of the quality of life (QOL) used for making the SEQOL (self-evaluation of quality of life) questionnaire or the quality of life as realizing life potential. This theory is strongly inspired by Maslow and the review furthermore serves as an example on how to fulfill the demand for an overall theory of life (or philosophy of life), which we believe is necessary for global and generic quality-of-life research.Whereas traditional medical science has often been inspired by mechanical models in its attempts to understand human beings, this theory takes an explicitly biological starting point. The purpose is to take a close view of life as a unique entity, which mechanical models are unable to do. This means that things considered to be beyond the individual's purely biological nature, notably the quality of life, meaning in life, and aspirations in life, are included under this wider, biological treatise. Our interpretation of the nature of all living matter is intended as an alternative to medical mechanism, which dates back to the beginning of the 20th century. New ideas such as the notions of the human being as nestled in an evolutionary and ecological context, the spontaneous tendency of self-organizing systems for realization and concord, and the central role of consciousness in interpreting, planning, and expressing human reality are unavoidable today in attempts to scientifically understand all living matter, including human life.

## INTRODUCTION

This paper presents one of the eight theories of the quality of life (QOL) used for making the SEQOL (self-evaluation of quality of life) questionnaire or the quality of life as realizing life potential. This theory is strongly inspired by Maslow[1,2] and has been presented in several publications[3,4]. The present overview furthermore serves as an example on how to fulfill the demand for an overall theory of life (or philosophy of life), which we believe[5,6] is necessary for global and generic quality-of-life research.

Whereas traditional medical science has often been inspired by mechanical models in its attempts to understand human beings, this theory takes an explicitly biological starting point. The purpose is to take a close view of life as a unique entity, which mechanical models are unable to do. This means that things considered to be beyond the individual's purely biological nature, notably the quality of life, meaning in life, and aspirations in life, are included under this wider, biological treatise.

Trying to include such lofty qualities of life in a biological theory will probably raise the suspicion that these qualities are going to be reduced to something more primitive. We hope that this analysis will make it clear that this is not the case. The purpose is to show a respect for life rather than to reduce it to mere trivial mechanisms.

## THE HIERARCHY OF LIFE

What is life from a modern biological viewpoint? If we look at how life is organized, we will immediately have a picture of a pyramid before our eyes: an organization of hierarchies with levels of biological systems nestled in one another, from the tiniest molecule, cell, and organism, to ecosystems and the biosphere[3,7].

In recent decades the belief has arisen that the essence of this hierarchy of life is not any material substance but the information that organizes the hierarchy[8]. What interests us are the self-reproducing patterns that contain these particles of matter. They are of interest because they contain what is life in a unique sense and not just the few grams of hydrogen, carbon, and other elements found in every living organism.

Recent surveys stress the ability of the physical, chemical, and biological world to generate new patterns spontaneously, where hitherto there were none[9,10]. This process is known as self-organization and it has been an ongoing process since the “Big Bang”. This is how the planets and the galaxies came into existence, and life has developed in this manner over 4 billion years. During the evolutionary process, biological systems developed more and more complex hierarchies with organisms and ecosystems forming through mutual interaction with the physical-chemical environment.

Higher-level organisms, such as animals and human beings, are colonies of cells that have united to form ever more complex systems with more and more cells for the past billion years[11]. To uphold the unity of the organism, the cells constantly exchange information to achieve maximum adaptation and coordination. This is because the subsystems of the organism have to carry out certain functions: they monitor the development and health of the organism as a whole. This life-maintaining communication between cells is called the biological information system.

The biological information system keeps the organism together and is found in all living beings — one-celled organisms, animals, and plants. The system that monitors the regulation and communication, including the hormones, the nervous system, and the immune system, is also responsible for the overall creation of form known as ontogenesis: the creation of each individual, regeneration of damaged tissue, etc.

Through evolution, the biological data system in more complex animals has generated a nervous system that processes sensory input from the environment, ensuring that the body reacts at maximum potential. An image of the external world is contained at the site where all sensory data is processed. In humans, this forum is the brain. Consciousness can be seen as an emergent quality of the organism. This quality emerges at a sufficiently high degree of complexity in the biological information system.

The human brain contains representations of external reality in the form of abstract maps of reality containing detailed descriptions of the external and internal world and the human self (the ego). Using these maps, we guide ourselves in our inner lives as well as in our external physical and social environment; as a consequence, these maps determine our ability to function in the world. All things being equal, poor maps will create poor lives and good maps good lives. This is because good maps of reality enable us to acknowledge, internally, our potential as well as the rich opportunities of our external lives, to identify these when they arise and to make constructive choices throughout life. The good map becomes the bridge to the world and ensures the optimal balance between the deep dreams and opportunities in life.

Fig. 1 presents a holistic model of the human being. The brain contains the map of reality, which, in the figure, is symbolized by a triangle. The map may be in good or poor shape, reflecting the person's understanding of life, himself, and the surrounding world. The brain is located within the unity and serves this unity through an image of the world (the map) by connecting it to the external world.

The map stems from personal history and is thus related to reason — everything we have learned about reality. The unity is linked to our actual existence and our deep lives and therefore contains the history of a life (as it has developed), a history the human being only has access to intuitively.

The theory of realizing life potential tells us that, by nature, human beings are capable of living: able to love themselves and others and to connect to the external world by occupying themselves with activities they care about and that are needed in the world. The real problem in life is to weed out the wrong and the outdated ideas such that the map is evolving internally in a proper balance with the inner being as well as the external reality.

The unity of the human being is created through a complex interaction between all parts of the organism, even at the cellular level, symbolized by the smaller levels in the bottom circle of Fig. 1 and all the arrows. Likewise, the unity of the world is created by integrating all living organisms into the global ecosystem. Thus, understanding ourselves does not entail discovering our own ego, but rather discovering the nature of our relationship to the world.

## THE BIOLOGICAL POTENTIAL AND LIFE INTENTIONS

Each living organism contains a store of information that reflects the evolution of that particular species. In recent decades we have become accustomed to viewing this mass concentration of piled-up information as carried by the genes. However, it is now becoming increasingly clear that both the cell surrounding the DNA strands and the data attached to the organism play a significant role in the formation and further development of biological form.

Let us then move one step further and state that living organisms have biological potential as a result of evolution. If we combine this notion with the universal tendency towards self-organization, we can conclude that biological potential requires the realization of life potential.

This urge of the biological potential to realize itself is termed the *will to live*. All living organisms, from bacteria to humans, have a will to live: a self-organizing instinct, or urge, that realizes the biological potential. We do not wish to posit a metaphysical or parapsychological, let alone, new (or old) life force beyond scientific description. Rather, we wish to give an intuitively plausible name to the tendency towards realization, a tendency that is no more mysterious than

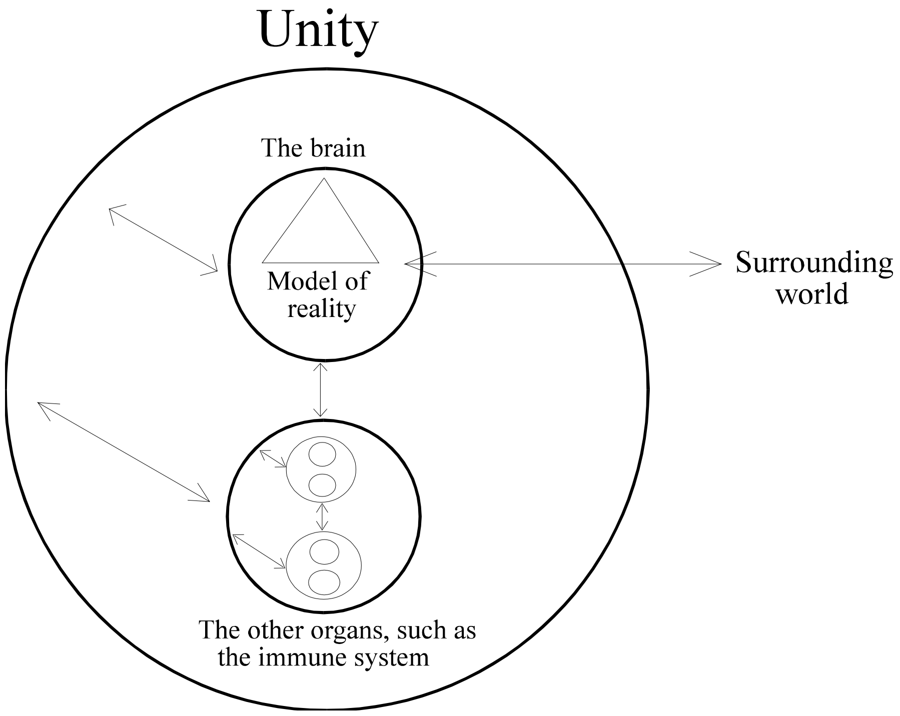

**FIGURE 1.** The wholeness of human being (see text for explanation).

that tendency towards self-organization, spontaneous order, and evolution that modern physics and biology have identified in numerous systems[12,13].

In humans, the will to live is expressed by biological potential: in physical evolution from the fertilized ovum to the mature body as well as in all the psychological and social activities human life requires when lived to the full.

We seldom come across the will to live per se in our everyday lives. Rather we see its actual manifestations in life, which we will call *life intentions or life purposes*[14]. Life intentions are the images of the present and the future that serve to give our lives the course we want them to take: the desire for a meaningful occupation, good social relations, family and children, stability or variety of life. As symbol-carrying images, often unconscious, life intentions form part of the values of life in the model of reality found in the brain. They are a part of our mental maps. As such they can further or inhibit the realization of life potential to varying degrees.

Life intentions are dormant seeds existing as dispositions of biological potential, dispositions that may shape the potential of a life. Nevertheless, our life intentions are really molded in close encounters with culture, especially our parents or other caregivers.

Our life intentions determine our efforts to develop life in certain directions. They are frustrated when we do not succeed in realizing life in accordance with our intentions. When this happens and we cannot reshape our life intentions, we tend to give them up and adapt the realization of life potential to the reality we find — we dream less ambitious dreams.

We see the will to live when we meet death. It is a powerful experience, because we are confronted with life, its intentions, and the basic urge to live. When we meet death, one of the major crises in life, we have to discover whether we are sufficiently strong to re-evaluate our true intentions and way of life. We may even have to change our attitude to it entirely. This in itself may lead to new growth and a reassessment of our values.

## ILL HEALTH AND MEANING IN LIFE

In the search for causes of ill health, medical science tends to examine genetic reasons such as defective molecules that cause malfunction or external stresses that cause traumas, such as traffic accidents, asbestos, or smoking. Only certain disciplines, notably psychosomatic medicine, pediatrics, and public health, are concerned with psychosocial factors.

Hence, medical science tends to believe that mechanical faults are the most important factors in the cause of ill health. Nevertheless, in the vast psychosocial field, the factors of the quality of life, may well be significantly more important than genetics and external stress factors. It could well be that the quality of life, and its many dimensions, is the major reason for ill health.

This is difficult to comprehend because consciousness and our entire worldview contribute to creating the quality of life. Life is far greater and more spacious than our perception of being and reality. As evolution concerns everything, not just you and me, the biological information system is a collective system, which, like the hierarchy of life, encompasses all individuals, including the biosphere. This link with the world plays a major role in the realization of life potential of each organism.

If we view human beings as organisms with biological potential capable of realizing themselves mentally and socially, the purpose of life is the ability to let this potential blossom and develop in an individual and ecosocial context. We may take this one step further so that the quality of life, a good life, means the ability to maximize life potential in a social and ecological context.

The acknowledgment of this and the ability to choose a good course in life (constantly adjusting one's life intentions in order to achieve the full realization of life potential) leading to a close connection to reality generate meaning in life. Meaning in life arises when we experience a fruitful connection between the inner depths of our being and the external world. The given biological potential is then realized and a unified worldview is hereby developed. This unification or connection may be experienced as stages reached in the realization of life (that is, manifest results such as having a partner and children) or it may take shape as a feeling that things are happening, that development is taking place in which the different phases in life are explored and seized gradually.

The experience of meaning in life presupposes a high degree of contact with the depths of our being, the center of our existence, which we here call the biological information system.

Meaning in life and hence the quality of life caused by our biological potential is how biology expresses itself subjectively in our lives. An objective expression of biological potential is found in our state of health. A healthy body is proof that the biological potential is finding a healthy outlet. Likewise, illness means that the biological potential is hampered in realizing itself in a healthy body. The biological information system, which is responsible for the biological potential, is out of balance in the sick body. Hence, communication between the cells, which is crucial for the maintenance of the organism as a whole, is disturbed.

A good example of this is cancer. Cancer is usually regarded as an illness in which the ability of the cells to fit into the organizational unity breaks down. This is the inevitable consequence of a breakdown in the mutual communication, regulation, and coordination of the cells.

Likewise, many other illnesses can be seen as a communication failure at an elementary, cellular level or as problems in the biological information system. This is further complicated when the immune system is affected. Infection, allergy, eczema, insulin-dependent diabetes, arthritis, multiple sclerosis, and other ailments can be explained if we think of these illnesses as a result of malfunctioning of the immune system. According to our understanding, this disregulation is linked to disturbances in the global biological information system of the organism[15,16]. Unfortunately, this global and integrative biological information system has yet no satisfying scientific description, but we believe it to carry our consciousness.

The subjective meaning in life and objective state of health of the individual is then a common basis of the individual's inner being or the existential center. As the quality of life is closely linked with self-realization and the degree of meaning in life attained, the quality of life is closely linked with illness via the state of the biological feedback system. As our quality of life is enhanced or diminished by the way we live and our opportunities to realize our biological potential, changes in lifestyle and the realization of life potential will change the quality of life and state of health. The relationship between the quality of life and illness is that both originate from the realization of life potential, that is, the ability to live out our life intentions. Fig. 2 outlines the roles the quality of life and state of health play in life. Three types are shown:

1. *A good life,* with high quality of life and good health where the individual lives to the full to the very end.
2. *An average life,* where the life intentions, quality of life, and the other subjective factors are neither quite right nor quite hopeless. This generates a poorer state of health and might end with thrombosis or cancer.
3. *A poor life*, where life intentions, quality of life, and maps of reality are bleak right from infancy. This leads to a life that is continuously downhill. Abandoning responsibility might lead to mental illness in which the individual no longer wishes to take part in collective reality.

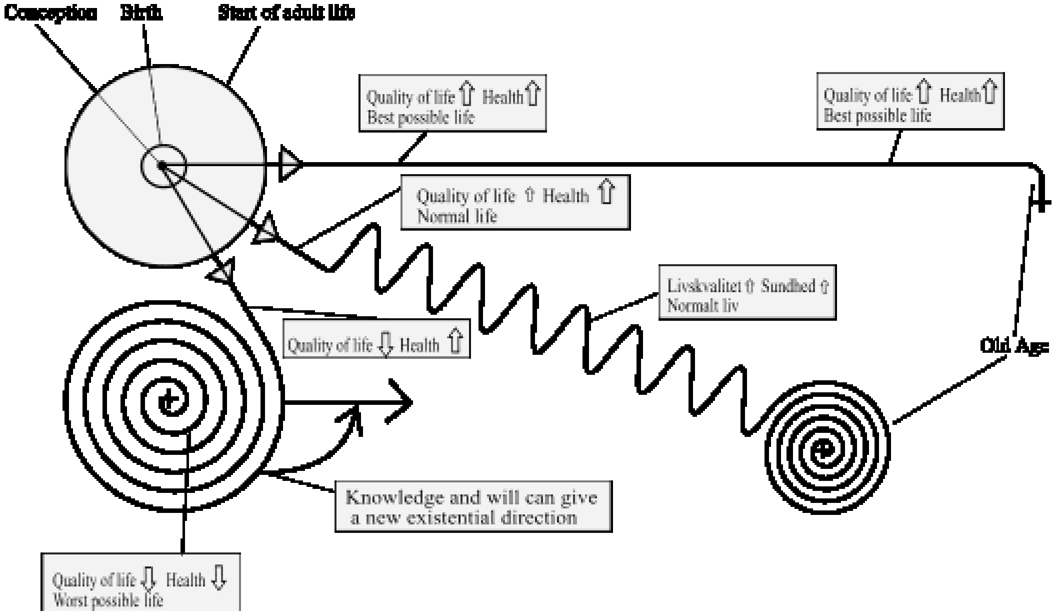

**FIGURE 2**. The three paths of life[4]: the good life with optimal QOL, health and ability of functioning, the average, normal, life with sub-optimal QOL, health and ability, and the poor life with low QOL, ill health and bad functioning, lived by a quartile of the Danish population.

We may choose to work with our fundamental notions of life and reality at any time. We can then adjust our course in life and thus achieve a better life[4,17,18,19]. Usually we need to meet death before we accept that we are no experts in life.

## TOWARDS A NEW SCIENTIFIC UNDERSTANDING OF HUMANITY

This understanding of the realization of life potential has many traits of a popular understanding of life and may strike us as being self-evident. Unfortunately, this is not the case. Another widely accepted model for the life and health of the human being, the medical science model, states in its biomedical mechanism that the cause of illness is either genes or external trauma: accidents, infections, bacteria, and other attacks on the body. The entire psychosocial field, including the mental maps of reality and life intentions, regarded in our model to be factors of affecting the quality of life, are not regarded as particularly meaningful in classical medical, molecular biology research. In fact, these factors are merely considered to be the background against which the factors causing the illness unfold and they are thus often ignored in scientific discussions of sickness and health.

The most important scientific hypothesis of our quality-of-life project is precisely that the quality of life, in the truest and deepest meaning of the concept, is the real cause of most illnesses, notably cancer, cardiovascular diseases, and allergies, and that these illnesses can be prevented by improving the quality of life in time[19]. If this hypothesis is confirmed and/or more explicitly formulated, it can lead to a new and far more comprehensive discipline of medicine than the one we know today.

Our interpretation of the nature of all living matter is intended as an alternative to medical mechanism, which dates back to the beginning of the 20th century. New ideas such as the notions of the human being as nestled in an evolutionary and ecological context, the spontaneous tendency of self-organizing systems for realization and concord, and the central role of consciousness in interpreting, planning, and expressing human reality are unavoidable today in attempts to scientifically understand all living matter, including human life.

## ACKNOWLEDGMENTS

This study was supported by grants from The 1991 Pharmacy Foundation, as well as by supplementary grants from Goodwill-fonden, the JL-Foundation, E. Danielsen and Wife's Foundation, Emmerick Meyer's Trust, the Frimodt-Heineken Foundation, the Hede Nielsen Family Foundation, Petrus Andersens Fond, Wholesaler C.P. Frederiksens Study Trust, Else & Mogens Wedell-Wedellsborg's Foundation, and IMK Almene Fond. We gratefully acknowledge the critical scrutiny and expert linguistic assistance of Ib Ravn, Ph.D. The research was approved by the Copenhagen Scientific Ethical Committee under number (KF)V.100.2123/91.

## BIOSKETCHES

**Søren Ventegodt, MD,** is the Director of the Quality of Life Research Center in Copenhagen, Denmark. He is also responsible for a Research Clinic for Holistic Medicine in Copenhagen and is a popular speaker throughout Scandinavia. He has published numerous scientific or popular articles and a number of books on holistic medicine, quality of life, and quality of working life. His most important scientific contributions are the comprehensive SEQOL questionnaire, the very short QoL5 questionnaire, the integrated QOL theory, the holistic process theory, the life mission theory, and the Danish Quality of Life Research Survey, 1991–94 in cooperation with the University Hospital of Copenhagen and the late pediatric professor Bengt Zachau-Christiansen. E-mail: ventegodt@livskvalitet.org. Website: www.livskvalitet.org/

**Joav Merrick, MD, DMSc,** is Professor of Child Health and Human Development affiliated with the Zusman Child Development Center and Division of Community Health at the Ben Gurion University, Beer-Sheva, Israel and presently the Medical Director of the Division for Mental Retardation, Ministry of Social Affairs, Jerusalem and the Director of the National Institute of Child Health and Human Development. He has numerous publications in the field of child and human development, rehabilitation, intellectual disability, disability, health, welfare, abuse, advocacy and prevention. Dr. Merrick received the Peter Sabroe Child Award for outstanding work on behalf of Danish Children in 1985 and the International LEGO-Prize ("The Children's Nobel Prize") for an extraordinary contribution towards improvement in child welfare and well being in 1987. E-mail: jmerrick@internet-zahav.net. Website: www.nichd-israel.com

**Niels Jørgen Andersen, MSc,** Professor, Department of Innovation and Economic Organization, Norwegian School of Management. This department conducts research and provides teaching in central topics related to innovation, business development, management of global companies, business history, and economic organization. Research activities within the Department are related to four core subjects within the discipline: business history, cooperative organizations, business development and entrepreneurship, and finally studies of industries with a special focus on the electricity industry. He is also the dynamic chairman of the nonprofit organization Stiftelsen Holistisk Medisin Scandinavia, which aims to support the scientific development, research, and documentation of complementary and holistic medicine in Scandinavia. E-mail: niels.j.andersen@bi.no. Website: www.bi.no/users/fgl93013/
